# Das SCATTER-Projekt: Computerbasierte Simulation zur Unterstützung bei der strategischen Verlegung von Intensivpatienten

**DOI:** 10.1007/s00103-023-03811-3

**Published:** 2023-12-28

**Authors:** Janina Bathe, Hanna-Joy Renner, Sven Watzinger, David Olave-Rojas, Leonie Hannappel, Jan Wnent, Stefan Nickel, Jan-Thorsten Gräsner

**Affiliations:** 1 Institut für Rettungs- und Notfallmedizin, Campus Kiel und Campus Lübeck, Universitätsklinikum Schleswig-Holstein, Arnold-Heller-Str. 3, Haus 808, 24105 Kiel, Deutschland; 2 Institut für Operations Research – Diskrete Optimierung und Logistik, Karlsruher Institut für Technologie, Karlsruhe, Deutschland; 3 Fachgruppe Intensivmedizin, Infektiologie und Notfallmedizin (Fachgruppe COVRIIN), Fachgebiet ZBS 7 – Strategie und Einsatz, Koordination: Robert Koch-Institut, Berlin, Deutschland; 4 School of Medicine, University of Namibia, Windhoek, Namibia; 5 Klinik f. Anästhesiologie und Operative Intensivmedizin, Campus Kiel, Universitätsklinikum Schleswig-Holstein, Kiel, Deutschland

**Keywords:** Entscheidungsfindungsprozess, Simulationstool, Verlegungsstrategien, Kleeblattkonzept, Krisenbewältigung, Decision-making process, Simulation tool, Transfer strategies, Clover leaf concept, Crisis management

## Abstract

**Hintergrund:**

Der Bedarf für ein Konzept für die bundesweite strategische Verlegung von Intensivpatienten wurde durch die COVID-19-Pandemie („coronavirus disease 2019“: Coronavirus-Krankheit-2019; ausgelöst durch eine Infektion mit dem Virus SARS-CoV-2) deutlich. Trotz des eigens hierfür entwickelten Kleeblattkonzeptes stellt die Verlegung einer großen Anzahl von Intensivpatienten eine große Herausforderung dar. Mithilfe einer Computersimulation werden in dem Projekt SCATTER (*S*trategis*C*he P*AT*ien*T*env*ER*legung) Verlegungsstrategien für die Krisenbewältigung am Beispiel eines fiktiven Szenarios getestet und Empfehlungen entwickelt.

**Methode:**

Nach sorgfältiger Erhebung von Prozess- und Strukturdaten für innerdeutsche Intensivtransporte erfolgte die Programmierung der Computersimulation. Hier können auf diverse Parameter Einfluss genommen und unterschiedlichste Verlegungsszenarien erprobt werden. In einem fiktiven Übungsszenario wurden von Schleswig-Holstein ausgehend bundesweite Verlegungen simuliert und anhand verschiedener Kriterien beurteilt.

**Ergebnisse:**

Bei den bodengebundenen Verlegungen zeigte sich aufgrund der eingeschränkten Reichweite und in Abhängigkeit der gewählten Zielregion, dass meist nicht alle Patienten verlegt werden können. Luftgebunden lässt sich zwar eine höhere Anzahl von Patienten verlegen, dies ist jedoch oft mit zusätzlichen Umlagerungen verbunden, die ein potenzielles Risiko für die Patienten darstellen. Eine distanzabhängige luft- oder bodengebundene Transportstrategie führte in dem Übungsszenario zu identischen Ergebnissen wie der rein luftgebundene Transport, da aufgrund der großen Distanz stets der luftgebundene Transport gewählt wurde.

**Diskussion:**

Aus der Computersimulation können wichtige Erkenntnisse über verschiedene Verlegungsstrategien und Rückschlüsse auf die Realität gezogen und Empfehlungen entwickelt werden.

## Abstract (en)

**Background:**

The need for a concept for the nationwide strategic transfer of critical care patients in Germany was highlighted during the COVID-19 (coronavirus disease 2019) pandemic. Despite the cloverleaf concept developed specifically for this purpose, the transfer of large numbers of critical care patients represents a major challenge. With the help of a computer simulation, the SCATTER research project uses a fictitious example to test, develop, and recommend transfer strategies.

**Method:**

The simulation was programmed after collecting procedural and structural data on critical care transports within Germany. The simulation allows altering various parameters and testing different transfer scenarios. In a fictitious scenario, nationwide transfers starting from Schleswig-Holstein were simulated and evaluated using predetermined criteria.

**Results:**

In the case of ground-based transfers, it became apparent that, depending on the selected target region, not all patients could be transferred due to the limited range of ground-based vehicles. Although a higher number of patients can be transferred by air, this is associated with additional gurney changes and potential risk to the patient. A distance-dependent transport strategy led to the identical results as purely air-bound transport, since air-bound transport was always chosen due to the long distances.

**Discussion:**

The simulation can be used to develop recommendations and to draw important conclusions from different transfer strategies.

## Hintergrund

Kapazitätsengpässe bei der regionalen Patientenversorgung können durch den Ausfall kritischer Infrastruktur durch Naturgefahren (z. B. Hochwasserereignisse), Ressourcenknappheit oder infolge biologischer Gefahren (z. B. Pandemien) entstehen. Zu Beginn der COVID-19-Pandemie („coronavirus disease 2019“: Coronavirus-Krankheit-2019; ausgelöst durch eine Infektion mit dem Virus SARS-CoV-2) im Frühjahr 2020 zeigte der Blick nach Italien, wie schnell eine Region an ihre Versorgungsgrenze, insbesondere für Intensivpatienten, stoßen kann. Bilder aus Bergamo verdeutlichten die Notwendigkeit einer nationalen Verlegungsstrategie bei regionalen Überlastungssituationen. In Deutschland gab es für die bundesweite Verlegung von (vielen) Intensivpatienten bislang kein Konzept oder Erfahrungswerte. Daher erarbeiteten die Bundesländer gemeinsam mit der Arbeitsgemeinschaft der Obersten Landesgesundheitsbehörden, dem Arbeitskreis V der Ständigen Konferenz der Innenminister und -senatoren der Länder, Bundesministerien, Robert Koch-Institut und Fachvertretern der Fachgruppe Intensivmedizin, Infektiologie und Notfallmedizin (COVRIIN) das Kleeblattkonzept [1]. Deutschland wurde hierfür in 5 Regionen (Kleeblätter) aufgeteilt. Jedes Kleeblatt hat einen sogenannten Single Point of Contact (SPoC), der als Ansprechpartner dient und die Koordination innerhalb des Kleeblatts und im bundesweiten Kontext übernimmt. Nach Ausschöpfung der regionalen Verlegungsmöglichkeiten innerhalb des eigenen Kleeblatts können in einer Überlastungssituation überregionale Verlegungen zum Kapazitätsausgleich initiiert werden, um für jeden Patienten eine adäquate intensivmedizinische Behandlung zu gewährleisten.

Eine derartige Überlastungssituation zeichnete sich Ende 2021 in Deutschland ab. Am 23.11.2021 aktivierten die Kleeblätter Ost und Süd das Kleeblattkonzept. Daraufhin wurden innerhalb von 37 Tagen 115 Patienten aus überlasteten Regionen in Gebiete mit ausreichenden Intensivkapazitäten verlegt. Die Wetterlage in Bayern limitierte initial die Nutzung von Intensivtransporthubschraubern (ITH), was die ohnehin komplexe Planung erschwerte.

Bei der Verlegung von mehreren Intensivpatienten über große Distanzen sind viele Entscheidungen zu treffen und Besonderheiten zu beachten. Dieser komplexe Entscheidungsprozess kann durch die Anwendung von Computersimulationen vorbereitet und Erkenntnisse aus der Simulation können auf die Realität übertragen werden. Das Potenzial von Computersimulationen als Unterstützungstool für logistische Prozesse im Gesundheitswesen zeichnet sich zunehmend in verschiedenen Forschungsprojekten ab [2–7].

Bis 2021 gab es keine wissenschaftliche Auseinandersetzung mit dem Thema strategische Patientenverlegungen. Um künftig für vergleichbare Situationen, wie etwa die COVID-19-Pandemie, gerüstet zu sein, wurde das vom Bundesministerium für Gesundheit geförderte und am Institut für Rettungs- und Notfallmedizin des Universitätsklinikums Schleswig-Holstein durchgeführte Forschungsprojekt SCATTER aufgesetzt. Ziel von SCATTER ist die Entwicklung eines computerbasierten Simulationsmodells, um den Vergleich von Transportstrategien und die Entwicklung von Entscheidungsempfehlungen zu ermöglichen sowie die im Kleeblattkonzept beschriebenen Vorgänge zu optimieren.

In der vorliegenden Publikation werden anhand der Ergebnisse eines fiktiven Szenarios die Vorteile von computerbasierten Unterstützungstools im Rahmen von Patientenverlegungen beschrieben und diskutiert. Die Methodik wird detailliert geschildert, um sichtbar zu machen, wie realitätsnah die Simulation entwickelt wurde und an welchen Stellschrauben diese ausgeweitet werden kann. Die Ergebnisse werden anhand der festgelegten Transportstrategien präsentiert und diese anschließend im operativen Kontext diskutiert.

## Methode

Computerbasierte Simulationsmodelle bilden ein real existierendes oder geplantes System mit seinen Prozessen und Abhängigkeiten ab. Jeder Simulationsdurchlauf ist ein sogenanntes Experiment, in dem verschiedene Szenarien simuliert oder einzelne Parameter geändert werden können. Im Anschluss können die Ergebnisse der einzelnen Experimente miteinander verglichen und bewertet werden. Aus den Erkenntnissen der Simulation können bei realitätsnaher Modellierung Rückschlüsse gezogen werden, wie sich das reale System (z. B. alle an der strategischen Verlegung eines COVID-19-Intensivpatienten beteiligten Strukturen) in bestimmten Situationen verhält. Im Rahmen von SCATTER wurde ein Simulationsmodell für die bundesweite Verlegung von COVID-19-Patienten entwickelt und im Simulationsprogramm AnyLogic implementiert. Im Bereich der regelhaften Notfallrettung wurden bereits mehrere Simulationsmodelle beschrieben [8, 9], die teilweise der hier beschriebenen Modellierung zur Orientierung dienten. Allerdings erforderte insbesondere die Modellierung eines Wechsels von Transportmitteln (multimodaler Transportprozess) die Entwicklung eines eigenständigen Simulationsmodells. Auf die verwendete Methode der Simulation wird in dem Artikel von Renner et al. [10] näher eingegangen.

### Strukturdaten

Zunächst wurden Krankenhaus- und weitere Strukturdaten erhoben, die alle Krankenhäuser des DIVI-Intensivregisters (DIVI = Deutsche Interdisziplinäre Vereinigung für Intensiv- und Notfallmedizin e. V.), die Verfügbarkeit von Hubschrauberlandeplätzen bzw. Koordinaten nächstgelegener Landeplätze, eine Einordnung der Krankenhäuser nach Versorgungsstufen entsprechend bundeslandspezifischer Einteilungen sowie Notfallstufen und Planbettenanzahlen enthalten.

Die Erfassung der Transportmittel erfolgte durch Abfragen über die Bundesländer, Kleeblatt-SPoC und auf Bundesebene und wurde durch eine Online-Recherche ergänzt. Es wurden Intensivtransportwagen (ITW), ITH und Hubschrauber im Dual-Use (Primärrettung und Intensivverlegungen) eingepflegt. Neben dem Standort mit aufgenommen wurden Schichtzeiten (bei den Hubschraubern ggf. an Sonnenauf- und -untergang gekoppelt) und Spezifikationen der Fahrzeuge (z. B. Anzahl der Perfusoren, Sauerstoffvorrat, höchstes zugelassenes Patientengewicht).

### Prozessdaten

Ein Simulationsmodell besteht aus Teilschritten und Parametern, die steuern, wie Prozesse im Detail ablaufen. Die Teilschritte des Verlegungsprozesses sind z. B. Alarmierung des Transportmittels, Ausrücken oder Aufnahme des Patienten in der Quellklinik. Für die Erhebung dieser sogenannten Prozessdaten wurde 374 anonymisierte Protokolle von COVID-19-Intensivtransporten im Zeitraum 14.03.2020 bis 05.07.2021 ausgewertet. Für die Durchführung der Studie liegt ein positives Ethikvotum der Medizinischen Fakultät der Christian-Albrechts-Universität zu Kiel vor (AZ: D 461/21). Folgende Transportdaten gingen in die Modellierung der Simulation mit ein: Vorbereitung, Anfahrt, Übernahme des Patienten, Transport, Übergabe des Patienten, Desinfektion und Rückführung des Transportmittels. Da die dokumentierten Zeiten oft nicht eindeutig zuzuordnen waren und viele der Zeiträume mehrere Prozessschritte beinhalten, erfolgte darüber hinaus stichprobenartig für einzelne Intensivtransporte eine detaillierte Zeiterfassung (*n* = 14), um einzelne Teilschritte des Prozesses, wie die Dauer der Umlagerung oder des Übergabegesprächs, besser abbilden zu können.

Aus den Intensivtransportprotokollen wurden außerdem die Therapieparameter und Charakteristika der Patienten erfasst, z. B. Beatmung, Kreislaufunterstützung, Spritzenpumpenanzahl, Größe, Gewicht (soweit verfügbar). Der Einfluss bestimmter Charakteristika (z. B. Vorhandensein einer arteriellen Blutdruckmessung oder einer Thoraxdrainage) auf die Transportzeit, insbesondere auf die Übergabezeit, wurde ermittelt.

### Auswahl der Zielklinik

Die Strategien zur Auswahl der Zielklinik (immer außerhalb des eigenen Kleeblatts) wurden wie folgt festgelegt:

- Verlegung in das nächstgelegene Krankenhaus mit freien Kapazitäten,
- Verlegung in das Krankenhaus mit der größten Kapazität zum Zeitpunkt der Verlegung,
- Verlegung in das Versorgungscluster mit der größten Kapazität auf allen Ebenen (Kleeblatt/Bundesland/Versorgungscluster) zum Verlegungszeitpunkt,
- Verlegung in das Krankenhaus mit den größten prognostizierten Kapazitäten,
- Verlegung in das Versorgungscluster mit den größten prognostizierten Kapazitäten.

Anhand der Daten des DIVI-Intensivregisters wurden freie Intensivkapazitäten für COVID-19-Patienten in die Simulation mit aufgenommen und um Prognosedaten der Universität Freiburg [11] ergänzt. Die Einbeziehung der Prognosedaten ist wichtig, um nicht in eine Region zu verlegen, die in absehbarer Zeit ebenfalls in eine Überlastungssituation steuert.

### Transportstrategie

Auch für die Zuteilung der Transportmittel wurden verschiedene Strategien erarbeitet:

- bodengebundener Transport,
- luftgebundener Transport,
- Transport luft- oder bodengebunden, abhängig von der Distanz zwischen abgebender und aufnehmender Klinik.

Vor der Zuteilung eines Transportmittels prüft der Computeralgorithmus außerdem die Erreichbarkeit der Zielklinik innerhalb der Arbeitszeit sowie das Vorhandensein der notwendigen Ausstattung. Wird beispielsweise aufgrund der Arbeitszeiten in der Simulation kein geeignetes Transportmittel gefunden, so wird der betreffende Patient zunächst auf eine Warteliste gesetzt. Sobald nach Beendigung laufender Transporte ein geeignetes Transportmittel verfügbar ist, wird der Patient zeitverzögert verlegt. Es kann allerdings auch vorkommen, dass ein Patient auf Basis der definierten Verlegungsregeln in der Simulation nicht verlegt werden kann, da kein Transportmittel die Verlegung zur zuvor festgelegten Zielklinik durchführen kann. Die Strategien sind nicht als Vorschläge für endgültige, in der Form operationalisierbare Strategien zu verstehen. Sie sind zum einen zur Validierung der Simulation gedacht und sollen zum anderen erste Aufschlüsse über die Effekte unterschiedlicher Entscheidungsfaktoren liefern.

### Einflüsse in der Simulation

Im realen Einsatzgeschehen gibt es eine große Varianz für bestimmte Prozessschritte einer Verlegung, z. B. die Dauer des ärztlichen Übergabegesprächs. In der Simulation wird daher aus der Verteilung der erfassten Protokollzeiten durch eine Zufallsfunktion die Dauer der Teilschritte bestimmt.

Da das Simulationsmodell die Patienten nacheinander den entsprechenden Zielkrankenhäusern und Transportmitteln zuweist, ergibt sich für die an den ersten Stellen der Liste stehenden Patienten ein kürzerer Transportweg. Durch die automatisierte Sortierung der Patientenliste gemäß Horovitz-Index^1^ wird gewährleistet, dass für Patienten mit einer schlechteren Beatmungssituation kürzere Transportwege generiert werden.

### Exemplarischer Einsatz der Simulation

Für die exemplarische Darstellung der Möglichkeiten, die die Simulation bietet, wird ein fiktives Szenario angenommen, bei dem es in Schleswig-Holstein zu einer Überlastungssituation kommt. 50 Patienten sollen zum Kapazitätsausgleich verlegt werden, alle orientieren sich in ihren Merkmalen an den Verlegekriterien des Kleeblattkonzepts (Tab. 1, [12]). Für die Patienten werden folgende Parameter festgelegt: positiver endexspiratorischer Druck (PEEP) zwischen 10 cm H_2_O und 14 cm H_2_O, inspiratorische Sauerstoffkonzentration (FiO_2_) zwischen 0,3 und 0,7, 25 Patienten mit Horovitz-Index 100–200, 25 Patienten mit Horovitz > 200, 5 Patienten schwerer als 120 kg, 3 Patienten mit einer Thoraxdrainage und 10 dialysepflichtige Patienten.

**Table 1:** 

SARS-CoV‑2	Positiv
Beatmung	Invasiv (endotrachealer Tubus oder Trachealkanüle)
FiO_2_	< 0,8
PEEP	< 15 cm H_2_O
Beatmungssituation	Stabil seit mind. 24 h
ECMO	Keine
Horovitz	> 100 (in Rückenlage)
pCO_2_	< 80 mm Hg
pH	> 7,3
Kreislaufsituation	Stabil seit mind. 24 h
Katecholaminbedarf	In den letzten 12 h maximal um 10 µg/min gesteigert
Invasive Blutdruckmessung	Vorhanden
Gewicht	< 120 kg, sonst nach Rücksprache
Größe	< 190 cm, sonst nach Rücksprache
Thoraxdrainage	Maximal eine mit Sog
Rückenlage	> 8 h möglich
Therapiewunsch	Gegeben
Einverständnis vom Patienten oder Bevollmächtigtem	Gegeben

Die Bedingungen, die ein COVID-19-Intensivpatient erfüllen muss, um für eine strategische Verlegung in Betracht gezogen zu werden, sind sehr streng. Bestimmte Voraussetzungen müssen therapieseitig erfüllt werden (z. B. Instrumentierung mit Tubus/Trachealkanüle und arterieller Zugang, keine ECMO-Therapie). Beatmungs- und Kreislaufsituation müssen stabilisiert sein und Größe und Gewicht des Patienten gehen in die Wahl des geeigneten Transportmittels ein

*SARS-CoV‑2* Severe Acute Respiratory Syndrome Coronavirus Type 2, *FiO*_*2*_ inspiratorische Sauerstofffraktion, *PEEP* positiver endexpiratorischer Druck, *ECMO* extrakorporale Membranoxygenierung, *pCO*_*2*_ Kohlendioxidpartialdruck

Durch die unterschiedlichen Transportmittel- und Auswahlstrategien ergeben sich insgesamt 15 Experimente, die sich bezogen auf unterschiedliche Variablen vergleichen lassen (Tab. 2).

**Table 2:** 

		Anzahl verlegbare Patienten	Transportdistanz (km)	Transportdauer (h)	Umlagerungen
Nächstgelegenes Krankenhaus	Bodengebunden	50	Ø 358,75	Ø 7,01	Keine: 50 Pat.
			Min. 148,35	Min. 4,22	1: 0 Pat.
			Max. 501,11	Max. 9,53	2: 0 Pat.
	Luftgebunden	50	Ø 283,73	Ø 4,81	Keine: 5 Pat.
			Min. 119,39	Min. 2,78	1: 19 Pat.
			Max. 379,91	Max. 8,12	2: 26 Pat.
	Distanzabhängig ITW oder ITH/RTH	50	Ø 283,73	Ø 4,81	Keine: 5 Pat.
			Min. 119,39	Min. 2,78	1: 19 Pat.
			Max. 379,91	Max. 8,12	2: 26 Pat.
Größte freie Kapazität Krankenhaus	Bodengebunden	2	Ø 467,44	Ø 9,26	Keine: 2 Pat.
			Min. 456,66	Min. 8,34	1: 0 Pat.
			Max. 478,21	Max. 10,17	2: 0 Pat.
	Luftgebunden	16	Ø 514,77	Ø 6,1	Keine: 1 Pat.
			Min. 342,47	Min. 3,54	1: 7 Pat.
			Max. 650,55	Max. 8,66	2: 8 Pat.
	Distanzabhängig ITW oder ITH/RTH	16	Ø 514,77	Ø 6,1	Keine: 1 Pat.
			Min. 342,47	Min. 3,54	1: 7 Pat.
			Max. 650,55	Max. 8,66	2: 8 Pat.
Größte freie Kapazität Krankenhaus nach Ebene	Bodengebunden	0	–	–	–
	Luftgebunden	8	Ø 641,27	Ø 7,16	Keine: 0 Pat.
			Min. 599,2	Min. 6,32	1: 8 Pat.
			Max. 656,15	Max. 8,69	2: 0 Pat.
	Distanzabhängig ITW oder ITH/RTH	8	Ø 641,27	Ø 7,16	Keine: 0 Pat.
			Min. 599,20	Min. 6,32	1: 8 Pat.
			Max. 656,15	Max. 8,69	2: 0 Pat.
Größte prognostizierte freie Kapazität Krankenhaus	Bodengebunden	1	Ø 657,2	Ø 9,9	Keine: 1 Pat.
			Min. 657,2	Min. 9,9	1: 0 Pat.
			Max. 657,2	Max. 9,9	2: 0 Pat.
	Luftgebunden	18	Ø 498,15	Ø 6,12	Keine: 1 Pat.
			Min. 257,06	Min. 3,97	1: 10 Pat.
			Max. 636,73	Max. 8,44	2: 7 Pat.
	Distanzabhängig ITW oder ITH/RTH	18	Ø 498,31	Ø 6,12	Keine: 1 Pat.
			Min. 257,06	Min. 3,97	1: 10 Pat.
			Max. 636,73	Max. 8,44	2: 7 Pat.
Größte prognostizierte freie Kapazität nach Ebene	Bodengebunden	0	–	–	–
	Luftgebunden	8	Ø 641,27	Ø 7,16	Keine: 0 Pat.
			Min. 599,2	Min. 6,32	1: 8 Pat.
			Max. 656,15	Max. 8,69	2: 0 Pat.
	Distanzabhängig ITW oder ITH/RTH	8	Ø 641,27	Ø 7,16	Keine: 0 Pat.
			Min. 599,2	Min. 6,32	1: 8 Pat.
			Max. 656,15	Max. 8,69	2: 0 Pat.

Dargestellt sind die Ergebnisse der 15 Experimente, die sich aus 3 Transportstrategien für jeweils 5 Strategien für die Zuordnung der Zielklinik ergeben. Aufgeführt sind die verlegbare Anzahl der 50 Simulationspatienten sowie die durchschnittliche Transportstrecke und Transportdauer. Außerdem wird aufgeführt, für wie viele Patienten zusätzliche Umlagerungen aufgrund von Transportmittelwechseln notwendig werden

*ITW* Intensivtransportwagen, *ITH* Intensivtransporthubschrauber, *RTH* Rettungshubschrauber

### Kriterien für die Bewertung der Ergebnisse

Zunächst lässt sich ein Vergleich in Hinblick auf die Transportmittel durchführen, z. B. wie viele Transportmittel für welchen Zeitraum gebunden sind. Auch in Bezug auf die Kapazitätsentwicklung in den Krankenhäusern, abweichend von der initialen Prognose, ist eine Auswertung möglich. Die Transportbedingungen für die Patienten können ebenfalls verglichen werden, z. B. die maximale Transportdauer und Anzahl der notwendigen Umlagerungen. Zusätzliche Umlagerungen werden bei luftgebundenen Transporten angenommen, wenn das Krankenhaus nicht über einen eigenen Landeplatz verfügt. Befindet sich der Landeplatz mehr als 100 m, aber weniger als einen Kilometer vom Krankenhaus entfernt, wird von einem Zwischentransport mit einem kurzfristig verfügbaren Transportmittel ausgegangen (RTW, krankenhauseigenes Transportsystem), was jedoch mit einer Umlagerung verbunden ist. Bei einer Entfernung von über einem Kilometer wird ein verfügbarer ITW geplant.

## Ergebnisse

Die Ergebnisse für die unterschiedlichen Strategien für eine Zuordnung zu den Zielkliniken unter Berücksichtigung der Transportmittelart zeigen sich wie folgt:

### Verlegung in das nächstgelegene Krankenhaus mit freien Kapazitäten außerhalb des eigenen Kleeblatts

Bei den Transporten in das nächstgelegene Krankenhaus können alle 50 Simulationspatienten verlegt werden, sowohl boden- als auch luftgebunden bzw. bei distanzabhängiger Wahl des Transportmittels. Da beim gewählten Szenario Schleswig-Holstein das verlegende Bundesland ist, wird bei der distanzabhängigen Entscheidung stets der Hubschrauber vom Algorithmus ausgewählt, da alle Zielkliniken mehr als 150 km von der jeweiligen Quellklinik entfernt sind. So ergeben sich identische Ergebnisse für luftgebundenen und distanzabhängigen Transport. Die durchschnittliche Transportdauer beträgt 7,01 h bodengebunden (4,22–9,53 h) bei einer Transportdistanz von durchschnittlich 358,75 km (148,35–501,11 km). Luftgebunden findet sich eine durchschnittliche Transportdauer von 4,81 h (2,78–8,12 h) bei einer Distanz von 283,73 km (119,39–379,91 km). Die kürzeren Distanzen ergeben sich durch die unterschiedlichen Streckenberechnungen (Luftlinie bzw. Straßenverlauf). Bei den luftgebundenen Transporten kommt es bei 19 Patienten zu einer und bei 26 Patienten zu 2 zusätzlichen Umlagerungen.

### Verlegung in das Krankenhaus mit den größten freien Kapazitäten außerhalb des eigenen Kleeblatts

Bei der Verlegung in das Krankenhaus mit der aktuell größten freien Kapazität zeigt sich für die Verlegungsstrategie mit Hubschraubern eine höhere Reichweite und daher die Möglichkeit, von den 50 Simulationspatienten insgesamt eine höhere Anzahl zu verlegen (16 luftgebundene im Vergleich zu 2 bodengebundenen Verlegungen). Allerdings ist hier auch ein größeres Risiko für Transporttraumata anzunehmen, da die Patienten häufiger umgelagert werden müssen (7 Patienten müssen zusätzlich einmal, 8 Patienten müssen zusätzlich 2‑mal umgelagert werden). Die durchschnittliche Transportdauer beträgt bodengebunden 9,26 h (8,34–10,17 h) und luftgebunden 6,1 h (3,54–8,66 h). Die durchschnittliche zurückgelegte Strecke beträgt bodengebunden 467,44 km (456,66–478,21 km) und luftgebunden 514,77 km (342,47–650,55 km).

### Verlegung in das Kleeblatt/Bundesland/Versorgungscluster mit der größten freien Kapazität

Mit einer Verlegungsstrategie, die nur bodengebundene Transporte zulässt und als Zielregion das Versorgungscluster mit der aktuell größten Kapazität avisiert, kann kein Transport disponiert werden. Die Distanz zwischen Quell- und Zielklinik ist zu groß, um sie innerhalb eines Arbeitstages zu überwinden. Luftgebunden können 8 Patienten verlegt werden mit einer durchschnittlichen Transportzeit von 7,16 h (6,32–8,69 h) über eine Distanz von durchschnittlich 641,27 km (599,2–656,15 km). Alle 8 Patienten müssen einmal zusätzlich umgelagert werden.

### Verlegung in das Krankenhaus mit der größten prognostizierten Kapazität

Wird die prognostizierte freie Kapazität betrachtet, so können 18 Patienten luftgebunden und nur einer bodengebunden verlegt werden. Der bodengebundene Transport erfolgt über eine Strecke von 657,2 km in 9,9 h. Der durchschnittliche luftgebundene Transport dauert 6,12 h (3,97–8,44 h) über eine Strecke von durchschnittlich 498,15 km (257,06–636,73 km). Bei der luftgebundenen Transportstrategie müssen 10 Patienten zusätzlich einmal und 7 Patienten zusätzlich 2‑mal umgelagert werden.

### Verlegung in das Kleeblatt/Bundesland/Versorgungscluster mit der größten prognostizierten freien Kapazität

Da auf Versorgungsclusterebene die größte prognostizierte freie Kapazität im gleichen Versorgungscluster zu finden ist wie zum betrachteten Verlegungszeitpunkt, entsprechen die Ergebnisse denen unter „Verlegung in das Kleeblatt/Bundesland/Versorgungscluster mit der größten freien Kapazität“ genannten.

**Figure 1:**
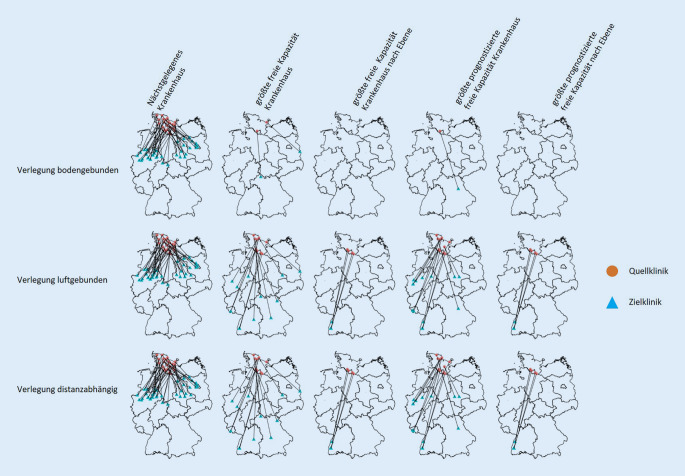

Die Abb. 1 zeigt die Verlegungen der Quell- und Zielkliniken für alle Verlegungsstrategien und alle Szenarien. Tab. 3 zeigt sowohl die Anzahl der an den Verlegungen beteiligten Strukturen (Kleeblätter, Bundesländer, Versorgungscluster) als auch den Einfluss der Verlegungen auf freie Kapazitäten in diesen Bereichen. Hierbei zeigt sich z. B. bei bodengebundenen Verlegungen in das nächstgelegene freie Krankenhaus unter Berücksichtigung der Prognosen und der Veränderung durch die aufgenommenen Patienten eine Überlastung auf Versorgungsclusterebene.

**Table 3:** 

		Anzahl Beteiligter		Max. aufgenommene Pat.		Max. relative Reduktion		Min. Restkapazität	
Nächstgelegenes Krankenhaus	Bodengebunden	Versorgungscluster (VC)	7	VC	27	VC	(^a^)	VC	−5
		Bundesländer (BL)	5	BL	27	BL	0,56	BL	8
		Kleeblätter (KL)	3	KL	27	KL	0,15	KL	113
	Luftgebunden	Versorgungscluster (VC)	7	VC	26	VC	(^a^)	VC	−5
		Bundesländer (BL)	5	BL	26	BL	0,61	BL	7
		Kleeblätter (KL)	3	KL	26	KL	0,16	KL	112
	Distanzabhängig ITW oder ITH/RTH	Versorgungscluster (VC)	7	VC	26	VC	(^a^)	VC	−5
		Bundesländer (BL)	5	BL	26	BL	0,61	BL	7
		Kleeblätter (KL)	3	KL	26	KL	0,16	KL	112
Größte freie Kapazität Krankenhaus	Bodengebunden	Versorgungscluster (VC)	2	VC	1	VC	(^a^)	VC	−1
		Bundesländer (BL)	2	BL	1	BL	0,05	BL	18
		Kleeblätter (KL)	2	KL	1	KL	0,01	KL	94
	Luftgebunden	Versorgungscluster (VC)	9	VC	3	VC	(^a^)	VC	−2
		Bundesländer (BL)	6	BL	4	BL	0,14	BL	17
		Kleeblätter (KL)	4	KL	7	KL	0,04	KL	91
	Distanzabhängig ITW oder ITH/RTH	Versorgungscluster (VC)	9	VC	3	VC	(^a^)	VC	−2
		Bundesländer (BL)	6	BL	4	BL	0,14	BL	17
		Kleeblätter (KL)	4	KL	7	KL	0,04	KL	91
Größte freie Kapazität Krankenhaus nach Ebene	Bodengebunden	Versorgungscluster (VC)	0	VC	0	VC	–	VC	–
		Bundesländer (BL)	0	BL	0	BL	–	BL	–
		Kleeblätter (KL)	0	KL	0	KL	–	KL	–
	Luftgebunden	Versorgungscluster (VC)	1	VC	8	VC	0,24	VC	26
		Bundesländer (BL)	1	BL	8	BL	0,09	BL	76
		Kleeblätter (KL)	1	KL	8	KL	0,04	KL	207
	Distanzabhängig ITW oder ITH/RTH	Versorgungscluster (VC)	1	VC	8	VC	0,24	VC	26
		Bundesländer (BL)	1	BL	8	BL	0,09	BL	76
		Kleeblätter (KL)	1	KL	8	KL	0,04	KL	207
Größte prognostizierte freie Kapazität Krankenhaus	Bodengebunden	Versorgungscluster (VC)	1	VC	1	VC	0,5	VC	1
		Bundesländer (BL)	1	BL	1	BL	0,01	BL	94
		Kleeblätter (KL)	1	KL	1	KL	0,01	KL	94
	Luftgebunden	Versorgungscluster (VC)	6	VC	6	VC	1	VC	0
		Bundesländer (BL)	5	BL	6	BL	0,18	BL	6
		Kleeblätter (KL)	4	KL	8	KL	0,04	KL	93
	Distanzabhängig ITW oder ITH/RTH	Versorgungscluster (VC)	6	VC	6	VC	1	VC	0
		Bundesländer (BL)	5	BL	6	BL	0,18	BL	6
		Kleeblätter (KL)	4	KL	8	KL	0,04	KL	93
Größte prognostizierte freie Kapazität nach Ebene	Bodengebunden	Versorgungscluster (VC)	0	VC	0	VC	–	VC	–
		Bundesländer (BL)	0	BL	0	BL	–	BL	–
		Kleeblätter (KL)	0	KL	0	KL	–	KL	–
	Luftgebunden	Versorgungscluster (VC)	1	VC	8	VC	0,24	VC	26
		Bundesländer (BL)	1	BL	8	BL	0,09	BL	76
		Kleeblätter (KL)	1	KL	8	KL	0,04	KL	207
	Distanzabhängig ITW oder ITH/RTH	Versorgungscluster (VC)	1	VC	8	VC	0,24	VC	26
		Bundesländer (BL)	1	BL	8	BL	0,09	BL	76
		Kleeblätter (KL)	1	KL	8	KL	0,04	KL	207

Dargestellt ist der Einfluss der 15 Experimente (3 Transportstrategien und 5 Strategien zur Festlegung der Zielklinik) auf die Auslastung der Intensivbettenkapazitäten auf den verschiedenen Ebenen (Versorgungscluster, Bundesland und Kleeblatt)

Abgesehen von der Anzahl der beteiligten Versorgungscluster, Bundesländer und Kleeblätter wird die maximale Anzahl der aufgenommenen Patienten dargestellt sowie die relative Reduktion der freien Kapazitäten, die sich dadurch ergibt. Darüber hinaus wird die minimale Restkapazität auf den Intensivstationen in den Bereichen aufgeführt

*ITW* Intensivtransportwagen, *ITH* Intensivtransporthubschrauber, *RTH* Rettungshubschrauber

^a^Nicht berechenbar, da mindestens eine historische Kapazität gleich 0 ist

## Diskussion

Der bundesweite strategische Transport von einer Vielzahl von Patienten ist herausfordernd. SCATTER ermöglicht die Erprobung verschiedener Szenarien und Transportstrategien unter Berücksichtigung vieler Informationen, z. B. der Prognose der Intensivkapazitäten in der Zielregion.

Die einzelnen Komponenten des Intensivtransports werden in der Computersimulation berücksichtigt: Die Darstellung der Krankenhauslandschaft ist so realitätsnah wie möglich gehalten, um eine Übertragbarkeit sicherzustellen. Es erfolgte die Erfassung der Notfallstufen und der Versorgungsstufen, womit weitestgehend gewährleistet wird, dass die Verlegung auf horizontaler Ebene stattfindet. Diese ist charakteristisch für den strategischen Aspekt – sie findet nicht aufgrund individualmedizinischer Notwendigkeit statt, sondern aufgrund einer (drohenden) Überlastung des regionalen Systems und zum Kapazitätsausgleich.

Die Wahl des geeigneten Transportmittels ist für einen erfolgreichen Intensivtransport wichtig [13, 14]. Gerade bei den bundesweiten Verlegungen sind ITW aufgrund langer Transportdauer oft nicht geeignet bzw. es müssen Faktoren wie der Sauerstoffbedarf berücksichtigt werden. Aber auch eine Verlegungsstrategie, die nur ITH-Transporte vorsieht, ist nicht Erfolg versprechend und in der Realität nicht immer umsetzbar. Bei den Kleeblattverlegungen waren im Winter 2021 während der Akutphase Verlegungen mittels Hubschrauber in Bayern wetterbedingt nicht möglich. Wetterszenarien, in denen in einem Gebiet keine luftgebundenen Verlegungen möglich sind, lassen sich in die Simulation einspielen.

Eine Verlegung mittels Hubschrauber, insbesondere bei kurzen Strecken, bietet nicht immer einen Zeitvorteil [15]. Dies ist vor allem der Fall, wenn die Krankenhäuser (Quell- und/oder Zielklinik) keinen eigenen Hubschrauberlandeplatz besitzen und somit Zwischentransporte notwendig werden. Die Anzahl der Umlagerungen ist als Qualitätsmerkmal eines Intensivtransports zu betrachten, da z. B. bei Patienten mit einem akuten Atemnotsyndrom (ARDS) jeder Wechsel des Beatmungsgeräts als potenzielles (Transport‑)Trauma zu bewerten ist. Wenn auch in einigen Regionen bereits die Infrastruktur gegeben ist, dass der Patient auf der gleichen Trage in Hubschrauber und RTW transportiert werden kann und damit eine Umlagerung und ggf. der Wechsel des Beatmungsgerätes wegfällt, so ist das doch noch nicht in allen Gebietskörperschaften der Fall.

Insgesamt sind die Möglichkeiten der Verlegungsstrategien in der Simulation genauso vielfältig wie in der Realität. Bei Bedarf könnten sie problemlos noch um weitere Komponenten ergänzt werden, wie z. B. zusätzliche ITW, Hubschrauber oder neue Intensivtransportmittel (wie z. B. Rettungszüge). Auch der Auswahl der Patienten und deren unterschiedlichen Ansprüchen an das Transportmittel kann in der Simulation Rechnung getragen werden. Die Vorgaben für Eigenschaften der Intensivpatienten, die im Rahmen der COVID-19-Pandemie verlegt wurden, waren sehr restriktiv, sodass eine sehr homogene Patientengruppe entstand. Die Simulation ist jedoch so aufgebaut, dass nicht nur der individuelle Sauerstoffverbrauch auf die Transportdistanz ausgerechnet und mit den Sauerstoffreserven des geplanten Transportmittels abgeglichen werden kann, sondern auch Sondertransporte (z. B. mit einer extrakorporalen Membranoxygenierung, ECMO) geplant werden können.

Besonders aufschlussreich ist bei den vorliegenden Experimenten die Unterscheidung zwischen Auswahl der Krankenhäuser aufgrund der aktuellen Belegung und unter Berücksichtigung der Prognose. Während bei der Auswertung der Entwicklung im aufnehmenden Versorgungscluster bei Verlegungen auf Basis der aktuellen Lage im Verlauf eine Überlastungssituation angenommen wird (Kapazität-5), kommt es bei Verlegungsstrategien basierend auf den erwarteten Prognosen zu keiner weiteren Überlastungssituation.

Zukünftig kann diese Modellierung ausgeweitet werden und auch andere Lagen simulieren, wenn z. B. die regionalen Behandlungskapazitäten von Krankenhäusern überschritten werden oder nicht in normalem Maß zur Verfügung stehen. Beispiele dafür wären ein Terroranschlag mit vielen Verletzten, Naturkatastrophen, Ausfall von kritischen Infrastrukturen etc. Eine Ausweitung zur Entwicklung von Strategien für internationale Verlegungen ist ebenfalls denkbar.

Die Computersimulation bezieht sich auf die eigens erstellte Datenbank von Krankenhäusern und Transportmitteln. Eine unzureichende Datenlage, wie z. B. bei den Intensivtransportmitteln, führt unmittelbar zu einer Unschärfe der Simulation. Die Datenbanken sind jedoch jederzeit erweiter- und änderbar.

Die in diesem Beitrag beschriebene Methode ist ein kosteneffizienter Ansatz zur Erprobung von Strategien zur Krisenbewältigung und zur Stärkung der Bereitschaftsplanung im Gesundheitswesen. Bevor Ressourcen aktiviert und möglicherweise das Leben von Patienten riskiert werden, ermöglicht die Nutzung eines solchen Simulationstools die virtuelle Erprobung von Plänen und kann dabei unterstützen, strategische und weitsichtige Entscheidungen zu treffen. Der Einsatz von Simulationen als Entscheidungshilfe bei der strategischen Zuweisung von Patienten ist in der Literatur zu COVID-19 unterrepräsentiert [16], da dort der Schwerpunkt eher auf der Modellierung der Epidemiologie und der Intensivkapazität liegt [17]. SCATTER schließt diese Lücke zwischen der epidemiologischen COVID-19-Modellierung und dem Einsatz von Simulation bei der Katastrophenvorsorge. Simulation in Zusammenhang mit dem Katastrophenmanagement ist vergleichsweise neu, wird aber zunehmend als wichtiger Ansatz anerkannt. Sie ermöglicht allen Beteiligten Einblicke in das reale System und bietet eine Plattform, auf welcher Experimente durchgeführt werden können, die sonst nicht realisierbar wären [18]. SCATTER ist ein Entscheidungsinstrument, das zur Beantwortung von Fragen der Vorbereitung und Planung sowie der Ressourcenzuweisung genutzt werden kann.

In einer Weiterentwicklung der Simulation wäre die Umsetzung eines Optimierungsmodells denkbar. Dabei testet die Simulation in einem vorgegebenen Szenario verschiedene Verlegungsstrategien selbstständig, entscheidet anhand im Vorfeld definierter Parameter, welche Strategie am vorteilhaftesten wäre, und schlägt diese dem Anwender vor. Durch ein solches Optimierungsmodell könnte SCATTER in hochkomplexen Lagen als Entscheidungsunterstützungstool eingesetzt werden und so die Entscheidungsträger entlasten.

## Fazit

Computersimulationen können eine wichtige Entscheidungshilfe bei der Bewältigung von komplexen Situationen sein. Je mehr Faktoren auf ein Geschehen Einfluss nehmen und bei der Planung von Strategien bedacht werden müssen, desto eher scheint eine digitale Unterstützung sinnvoll. Mit der dargestellten Computersimulation lassen sich Verlegungsstrategien von Intensivpatienten in Hinblick auf Machbarkeit prüfen und die Auswirkung auf Patienten, Transportmittel und Intensivkapazitäten auf den unterschiedlichen Ebenen vergleichen.

## Footnotes

1 Horovitz-Index: Quotient aus arteriellem Sauerstoffpartialdruck und inspiratorischem Sauerstoffanteil, der in der Intensivmedizin zur Beschreibung der Lungenfunktion verwendet wird (je größer die Lungenschädigung desto niedriger der Wert).
